# Genetic Variants in Folate and Cobalamin Metabolism-Related Genes in Pregnant Women of a Homogeneous Spanish Population: The Need for Revisiting the Current Vitamin Supplementation Strategies

**DOI:** 10.3390/nu14132702

**Published:** 2022-06-29

**Authors:** Gemma Rodriguez-Carnero, Paula M. Lorenzo, Ana Canton-Blanco, Leire Mendizabal, Maddi Arregi, Mirella Zulueta, Laureano Simon, Manuel Macia-Cortiñas, Felipe F. Casanueva, Ana B. Crujeiras

**Affiliations:** 1 Epigenomics in Endocrinology and Nutrition Group, Epigenomics Unit, Instituto de Investigacion Sanitaria de Santiago de Compostela (IDIS), Complejo Hospitalario Universitario de Santiago de Compostela (CHUS/SERGAS), 15706 Santiago de Compostela, Spain; maria.gemma.rodriguez.carnero@sergas.es (G.R.-C.); paula.marino.lorenzo@sergas.es (P.M.L.); ana.canton.blanco@sergas.es (A.C.-B.); 2 Endocrinology and Nutrition Division, Complejo Hospitalario Universitario de Santiago de Compostela (CHUS/SERGAS), 15706 Santiago de Compostela, Spain; 3 CIBER Fisiopatologia de la Obesidad y Nutricion (CIBERobn), 28029 Madrid, Spain; felipe.casanueva@usc.es; 4 Patia Europe, 20009 San Sebastian, Spain; lmendizabal@patiadiabetes.com (L.M.); marregui@patiadiabetes.com (M.A.); mzulueta@patiadiabetes.com (M.Z.); lsimon@patiadiabetes.com (L.S.); 5 Gynecology and Obstetrics Division, Complejo Hospitalario Universitario de Santiago de Compostela (CHUS/SERGAS), 15706 Santiago de Compostela, Spain; manuel.macia.cortinas@sergas.es; 6 Molecular and Cellular Endocrinology Group, Instituto de Investigacion Sanitaria de Santiago de Compostela (IDIS), Complejo Hospitalario Universitario de Santiago de Compostela (CHUS/SERGAS), Santiago de Compostela University (USC), 15706 Santiago de Compostela, Spain

**Keywords:** vitamin B9, vitamin B12, pregnancy, newborn, polymorphism, one-carbon metabolism, MTHFR, MTR, CUBN, SLC10A1

## Abstract

Polymorphisms of genes involved in the metabolism and transport of folate and cobalamin could play relevant roles in pregnancy outcomes. This study assessed the prevalence of genetic polymorphisms of folate and cobalamin metabolism-related genes such as MTHFR, MTR, CUBN, and SLC19A1 in pregnant women of a homogeneous Spanish population according to conception, pregnancy, delivery, and newborns complications. This study was conducted on 149 nulliparous women with singleton pregnancies. Sociodemographic and obstetrics variables were recorded, and all patients were genotyped in the MTHFR, MTR, CUBN, and SLC10A1 polymorphisms. The distribution of genotypes detected in this cohort was similar to the population distribution reported in Europe, highlighting that more than 50% of women were carriers of risk alleles of the studied genes. In women with the MTHFR risk allele, there was a statistically significant higher frequency of assisted fertilisation and a higher frequency of preeclampsia and preterm birth. Moreover, CUBN (rs1801222) polymorphism carriers showed a statistically significantly lower frequency of complications during delivery. In conclusion, the prevalence of genetic variants related to folic acid and vitamin B12 metabolic genes in pregnant women is related to mother and neonatal outcomes. Knowing the prevalence of these polymorphisms may lead to a personalised prescription of vitamin intake.

## 1. Introduction

Pregnancy is a critical period during which a mother’s nutrition and lifestyle have a decisive influence on maternal and child outcomes. Multiple factors can affect pregnancy health, including maternal sociodemographic characteristics, environmental exposures, maternal nutrition, age, obesity, lifestyle, and socioeconomic status, as well as genetic background and gene–environment interactions [1,2,3]. 

The maternal diet and nutritional stores provide nutrients for the developing embryo and foetus [4,5,6,7,8]. Among nutrients, folic acid (vitamin B9) and cobalamin (vitamin B12) stand out for their role in foetal growth and prevention of neural tube defects, through their role as essential co-factors in the one-carbon metabolism pathway [9,10,11]. Moreover, deficiencies in these vitamins were also associated with important impacts on the health of mothers such as preeclampsia [12], gestational diabetes [13], or maternal neurocognitive symptoms [14]. Considering this issue, supplementation with folic acid was particularly recommended by the World Health Organisation (WHO) in order to prevent pregnancy outcomes [15]. However, maternal vitamin levels depend on dietary and supplement intake but also are influenced by genetic polymorphisms in the gene coding for enzymes involved in vitamin metabolism and transport, which may lead to changes in their catalytic activity. Functional polymorphisms of genes encoding enzymes involved in one-carbon metabolism can cause disturbances in B9 and B12 vitamin status due to a reduction in enzyme activity [16,17,18,19]. Polymorphisms of genes involved in the metabolism and transport of these vitamins were associated with disturbances in the health of mother and child [20]. 

In this regard, determining genetic variants in folate and cobalamin metabolism-related genes in pregnant women can lead to personalised treatment with higher amounts of folic acid and cobalamin for the sake of improving pregnancy and neonatal health outcomes. Therefore, the aim of the present study was to assess the prevalence of the common target genetic polymorphisms of folate and cobalamin metabolism-related genes in the literature such as methylenetetrahydrofolate reductase (*MTHFR*), methionine synthase (*MTR*), cubilin (*CUBN*), and *SLC19A1* (commonly known as reduced folate carrier (RFC1)) in pregnant women of a homogeneous Spanish population according to conception, pregnancy, delivery, and newborn complications.

## 2. Patients and Methods

### 2.1. Study Population and Design

This study was conducted on 149 nulliparous women with singleton pregnancies. Pregnant women were recruited in the Endocrinology and Obstetrics departments of the “Complejo Hospitalario Universitario de Santiago de Compostela (CHUS)” in Santiago de Compostela, Galicia (northeastern Spain) from September 2018 to February 2020. Women were recruited between 24 and 28 weeks of pregnancy and then followed up throughout the pregnancy to delivery. The inclusion criteria were an age of 16 years old or older, singleton pregnancy, lack of any chronic disease or being under medical treatment, absence of any language barrier, and correct monitoring of pregnancy and delivery in our centre.

### 2.2. Sociodemographic and Obstetrics Variables 

At the time of recruitment, the following data were collected: age; ethnicity; maternal lifestyles (tobacco smoking, alcohol consumption, or drug abuse) during the pregnancy; singleton or multiple pregnancies; previous miscarriages, abortions, or ectopic pregnancies; use of assisted reproductive technologies and medical history (hypertension, diabetes); maternal height; and self-reported pre-pregnancy weight and weight at week 36 (or last weight in the case of preterm delivery) of pregnancy, which allowed for calculation of weight gain during pregnancy and body mass index pre-pregnancy.

### 2.3. Vitamin B12 and Folic Acid Supplementation Use 

Information on vitamin supplementation was obtained by reviewing electronic medical records and asking pregnant women from recruitment to delivery. Information on supplement intake included brand name, dosage per day, and the start and end dates of consumption. This information was used to determine supplemental vitamin B12 and folate doses per day for each woman. 

### 2.4. Maternal and Neonatal Outcomes and Definitions 

Participants were followed prospectively from recruitment until delivery. Maternal and neonatal outcomes were collected from electronic medical records.

Maternal outcomes such as gestational diabetes mellitus (GDM), gestational hypertension (GHT), and preeclampsia were obtained. GDM was defined as diabetes diagnosed in the second or third trimester of pregnancy that was not clearly overt diabetes prior to gestation and was diagnosed according to our Hospital’s protocol and the one-step approach [21]. GHT was defined as blood pressure ≥140/90 mmHg arising after 20-week gestation, without any other feature of the multisystem disorder that resolves within 3 months postpartum [22]. PE was defined as GHT with ≥1 proteinuria/abnormal renal or liver function tests or platelet count/symptoms and signs consistent with end-organ damage of preeclampsia [22].

Gestational age and anthropometric measurements (weight, height, head circumference, and chest circumference) at birth were obtained from electronic medical records. Weight index was calculated as the ratio of birth weight (grams) to height (cm^3^). Size for gestational age was estimated based on Carrascosa et al. (2004) [23], using neonatal gestational age at delivery, anthropometric measurements (AM: weight, height, and head circumference), and sex. Newborns were categorised into three groups: small for gestational age (SGA) (AM less than 10th percentile for gestational age); appropriate for gestational age (AGA) (AM 10th to 90th percentile for gestational age, which was the reference group); and large for gestational age (LGA) (AM greater than 90th percentile for gestational age). For birth weight, newborns were considered to be LGA if their birth weight was >2.0 standard deviation (SD) or over the 90th percentile for sex and gestational age, and SGA infants were considered to have a birth weight that was <−2.0 SD or under the 10th percentile for sex and gestational age. Normal birth weight was considered for values between −2.0 and +2.0 SD for sex and gestational age (between the 10th and 90th percentile). Low birth weight (LBW) was defined as birth weight less than 2500 g. Macrosomia was defined as neonates whose birth weight was equal to or greater than 4000 g.

Other obstetric complications such as miscarriage, stillbirth, and neonatal death were also collected. Delivery data were obtained, with special attention to the type of delivery (spontaneous onset of labour, induced labour, instrumental delivery, and route of delivery), preterm delivery, perinatal complications, admission to intensive care unit, and hospital stay. Spontaneous preterm birth (PTB) was spontaneous preterm labour or preterm premature rupture of the membranes, resulting in birth at <37-week gestation. Uncomplicated pregnancy was defined as a pregnancy in which no antenatal medical or obstetric complication had been diagnosed, resulting in the delivery of an appropriately grown, healthy baby at ≥37 weeks of gestation.

### 2.5. Sample Collection

Participants were asked to refrain from eating, drinking, brushing teeth, and using mouthwash for at least 30 min prior to sample collection for which a buccal swab of the Puritan Medical Products PurFlock Ultra^®^ (25-3606-U, Guilford, NC, USA) was used. Genomic DNA was extracted from the buccal swab using a MagMax DNA Multi-Sample Ultra Kit (Applied biosystems by Thermo Fisher Scientific, Waltham, MA, USA).

### 2.6. Genotyping

Genotyping was performed via IPLEX MassARRAY PCR using the Agena platform (Agena Bioscience, San Diego, CA, USA). IPLEX MassARRAY PCR and extension primers were designed from sequences containing each target SNP and 150 upstream and downstream bases with Assay Designer 4.0.0.2 (Agena Bioscience, San Diego, CA, USA) using the default settings. Single base extension reactions were performed on the PCR reactions with the iPLEX Gold Kit (Agena Bioscience) and 0.8 µL of the custom UEP pool. PCR reactions were dispensed onto SpectroChipArrays with a Nanodispenser (Agena Bioscience). An Agena Bioscience Compact MassArray Spectrometer was used to perform MALDI–TOF mass spectrometry according to the iPLEX Gold Application Guide. The Typer 4 software package (Agena Bioscience) was used to analyse the resulting spectra, and the composition of the target bases was determined from the mass of each extended oligo. These panels were designed in collaboration with PATIA, and genotyping was performed using the Agena platform located at the Epigenetics and Genotyping laboratory, Central Unit for Research in Medicine (UCIM), Faculty of Medicine, University of Valencia, Valencia, Spain.

### 2.7. Statistics Analysis

For the statistical analysis, 136 of the 149 patients were included, for whom genetic data were available. Different statistical tests were applied to examine the association between polymorphism in CUBN, MTHFR, MTR, and SLC19A1 and maternal and infant phenotypes and delivery complications. Maternal complications included preeclampsia, hypertension, and a family history of type 2 diabetes. Delivery complications included caesarean birth and induced birth. Neonatal phenotypes included macrosomia, admission to the intensive care unit, and PTB. The SNP information was decoded to numerical type depending on the presence of a reference or an alternative nucleotide of each sample. *p* values were computed using the chi-square test to determine whether the prevalence of genotype risk groups varied significantly depending on the phenotype or complication. *p* ≤ 0.05 was considered statistically significant. All of the aforementioned statistical analyses were performed using R software (version 3.2.0).

## 3. Results

### 3.1. Baseline Characteristics

Table 1 describes the characteristics of participants in this study (*n* = 149), including vitamin supplementation in the first trimester and neonatal and maternal outcomes. The mean age was 34.7 ± 5.2 years old, and the mean pregestational BMI was 26.4 ± 5.50 kg/m^2^; the average weight gain at final of pregnancy was 11.6 ± 5.84 kg. Spontaneous gestation was observed with a higher frequency than assisted. Delivery was spontaneous in the majority of patients. Regarding maternal complications during gestation, 62.4% had GDM, 2.0% GHT, and 3.4% preeclampsia. Regarding neonatal outcomes, the mean birth weight was 3.18 ± 0.57 kg, 7.4% were PTB, and 8.8% were admitted to intensive care.

All women received folic acid supplementation in the first trimester of pregnancy; 81.2% received a multivitamin complex that included folic acid, iodine, and B12; 13.4% received folic acid plus iodine; and 5.4% just folic acid. In addition to the supplementation with folic acid or multivitamin complex, 55.0% of women were prescribed iron supplementation.

### 3.2. Distribution of SNP Genotypes of Genes Related to Folate and Cobalamin Metabolism and Transport in the Study Population

**Figure 1:**
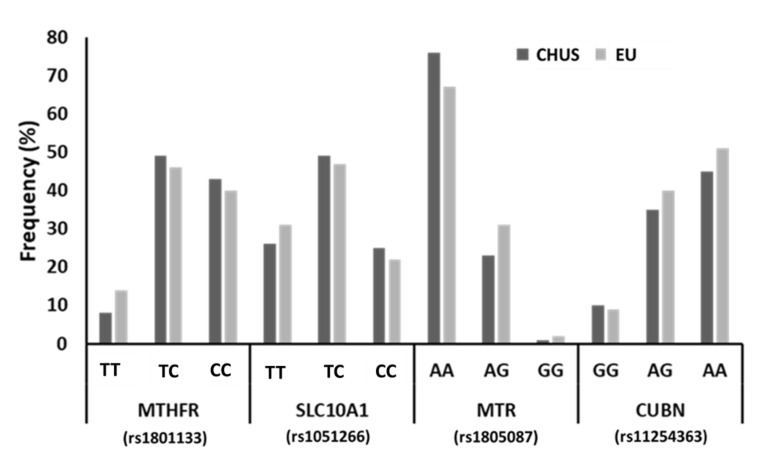
Prevalence of SNP genotypes of genes related to folate and cobalamin metabolism and transport in the study population (CHUS), compared with European population (EU). AA, adenine-adenine genotype; AG, adenine-guanine genotype; GG, guanine-guanine genotype; TT, thymine-thymine genotype; TG, thymine-guanine genotype; CC, cytosine-cytosine genotype; TC, thymine-cytosine genotype; MTHFR, methylenetetrahydrofolate reductase; SLC10A1, reduced folate carrier (RFC1); MTR, methionine synthase; CUBN, cubilin.

The distribution of genotypes detected in women from the CHUS cohort was similar to the population distribution reported in Europe (Figure 1). It is important to highlight that 61% of the women analysed in this study carried risk alleles in the MTHFR gene, approximately 72% of women in this study carried risk alleles in the SCL19A1 gene, and risk alleles were detected in the CUBN gene in 47% of these women, while the majority of this population carried the reference allele for the MTR gene (99%).

### 3.3. Association between SNP Genotypes and Pregnancy Outcomes

The association between maternal SNP and neonatal and maternal outcomes is shown in Table 2. Although only 1% of women were risk genotype carriers of *MTR*, and 75% of women in this study carried risk alleles in the *SCL19A1* gene, statistically significant associations were not found between the polymorphisms analysed of these genes, nor in *CUBN* and maternal or neonatal outcomes.

In women with the *MTHFR* risk allele, there was a statistically significant higher frequency of assisted fertilization (lower frequency of spontaneous gestation). In addition, there was a higher frequency of preeclampsia and PTB in women with variation in the genotype of *MTHFR*.

**Figure 2:**
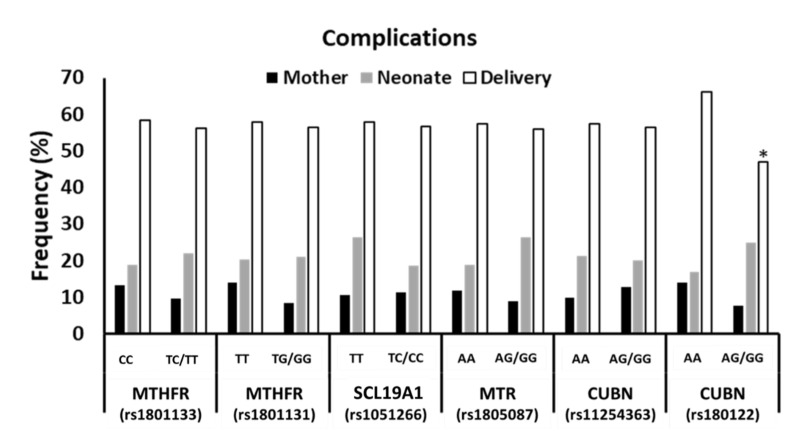
Association between complications in health of the mother, that of the neonate, or during delivery and SNP genotypes of genes related to folate and cobalamin metabolism and transport in the study population. (*) indicates statistically significant differences in delivery complications (white bars) between both groups of genotypes (AA vs. AG/GG). AA, adenine-adenine genotype; AG, adenine-guanine genotype; GG, guanine-guanine genotype; TT, thymine-thymine genotype; TG, thymine-guanine genotype; CC, cytosine-cytosine geno-type; TC, thymine-cytosine genotype; MTHFR, methylenetetrahydrofolate reductase; SLC10A1, reduced folate carrier (RFC1); MTR, methionine synthase; CUBN, cubilin.

When the cohort was analysed according to complications in the mother’s health, neonate’s health, or delivery, no association was found between genotypes and complications except for *CUBN* (rs1801222), which showed a lower frequency of complications during delivery associated with the risk allele (white bars, *p* = 0.024; Figure 2).

## 4. Discussion

The present study was carried out on pregnant women from northwestern Spain, which showed diversity in the prevalence of one-carbon metabolism risk polymorphisms. Importantly, among the evaluated SNPs, those related to *MTHFR* were associated with a lower frequency of spontaneous gestation and higher frenquecy ofpreeclampsia, and PTB, while the *CUBN* polymorphism was associated with a lower frequency of complications during delivery. Detection of risk alleles in women may lead to personalised medicine with targeted treatment based on increased vitamin intake that can improve success in conception and maternal and foetal outcomes.

Different genetic variants have been related to vitamin deficiency. The population prevalence of most of the polymorphisms associated with altered vitamin levels is unknown. The most widely studied polymorphism of one-carbon metabolism is the *MTHFR* genotype 677 C > T (rs1801133), which is responsible for the synthesis of the *MTHFR* enzyme and whose activity is decreased in TT homozygosis (by 60%) and in CT heterozygosis (by 30%) with respect to the CC genotype [24]. The prevalence of the TT SNP, the highest risk genotype, represented between 14.2% and 19.9% [25,26] of the cases described in studies carried out in Europe. More recently, Aguilar-Lacasaña et al. (2021) showed a higher prevalence (38%) in pregnant women in southern Spain [27]. In the current study, the prevalence in pregnant women carriers of TT SNP was 57%. This alteration has great relevance because individuals with TT have lower levels of folic acid than those with CC and CT [28,29,30].

Several studies have shown that the homozygous TT genotype has a lower response to folic acid treatment than those with the homozygous CC genotype, suggesting that the TT genotype requires higher folic acid intake [31]. The impact of these genetic alterations on folic acid levels and their effect on different diseases [31,32] such as breast, lung, or colorectal cancer [33] is widely known. Specifically, in pregnant women, the homozygous TT genotype has been associated with an increased risk of neural tube defects [34,35,36,37], reinforcing the idea of the well-known link between folic acid deficiency during pregnancy and the risk of neural tube defects in the newborn.

In our study, all women received supplements with folic acid or folic acid and B12. In this regard, Colson et al. (2017) [38] showed that a dose of 400 mcg daily of folic acid would be sufficient to overcome the deficits resulting from these polymorphisms. However, a recent study by Aguilar-Lacasaña et al. (2021) [27] showed that, although their population had received a correct folic acid intake during gestation, the prevalence of SGA and LGA was higher in pregnant women with T or TT in relation to the hetero or homo polymorphism CC, suggesting that there must be other factors that influence these results.

In addition, different studies have shown that women with the *MTHFR* 677 TT genotype are predisposed to elevated homocysteine levels when folic acid intake is inadequate [39], endothelial damage, arterial constriction, and thrombosis [40,41], all of which can lead to placental hypoperfusion resulting in worse neonatal outcomes with PTB and LBW [42]. In our study, women with the *MTHFR* risk alleles had a higher frequency of PTB, even though they all received adequate folic acid, but no higher frequency of LBW was observed. In addition, this higher frequency of premature newborns is concomitant with a higher frequency of LBW, which is a frequent neonatal complication [43,44] that has a direct implication in adolescence and adulthood, with a higher prevalence of chronic diseases such as obesity, diabetes, metabolic syndrome, or cardiovascular pathology [45].

Elevated homocysteine levels, derived from the incorrect functioning of the *MTHFR* enzyme, have also been related to an increased risk of spontaneous abortions [46]. In our series, women with the *MTHFR* risk allele had a lower frequency of spontaneous pregnancy, i.e., a greater need for assisted fertilization, which may reflect a difficulty in gestation derived from the incorrect functioning of the one-carbon metabolism pathway. The *MTHFR* risk allele in our population was associated with a lower frequency of spontaneous pregnancy, which is known to have a negative impact on health care costs and maternal mental health.

In our series, women with risk alleles of *MTHFR* had a higher frequency of preeclampsia than those without. Preeclampsia is characterised by hypertension accompanied by proteinuria in pregnant women over 20 weeks of gestation. This disease can affect both the foetus and the mother and, in extreme situations, can compromise the life of both [47,48,49]. A meta-analysis report by Wang et al. (2013) [50] showed a significant association between the *MTHFR* T allele and pre-eclampsia among Caucasians and people of Asian descent but not among people of African descent. However, a recent study carried out in Lagos, southwestern Nigeria, has shown an occurrence of preeclampsia was significantly associated with the presence of the T allele of *MTHFR* (OR = 1.855; *p* < 0.05) [51].

As for the cubilin gene (*CUBN*), the intrinsic factor-vitamin B12 receptor, we found that the allelic distribution of SNP rs1801222 was significantly different depending on complications during delivery—namely, a higher frequency of delivery complications was found among carriers of the wild-type allele. This suggests that carrying this genetic variant could reduce the risk of delivery complications. In this regard, previous studies have observed a significant increase in the risk of congenital heart disease for carriers of the wild-type allele of the *CUBN* SNP rs11254363 [52]. Cubilin favours the absorption of intrinsic factor-vitamin B12 complex in the intestinal mucosa. Polymorphisms in the cubilin gene were associated with variability in the binding and transport of vitamin B12. In this regard, it was demonstrated in a meta-analysis that participants homozygous for the rs1801222 G allele had higher plasmatic B12 levels [53]. Adequate maternal vitamin B12 status is associated with advantageous maternal and child health outcomes [54]. Therefore, the low frequency of delivery complications observed in G carriers in the current study could be related to higher plasmatic B12 levels in these participants. As far as we know, this is the first study that found a different distribution of *CUBN* SNPs in women with delivery complications, such as caesarean section and induced or instrumental delivery.

Other relevant polymorphisms involved in the one-carbon metabolism are *MTR* and *SLC19A1*. *MTR* encoded an enzyme involved in the synthesis of methionine through homocysteine methylation with the presence of vitamin B12 (Vit. B12) as a co-factor, and SNPs in this gene were associated with risk during pregnancy [55]. SLC19A1 gene encodes a typical transporter with 12 transmembrane domains involved in the active transport of 5-methyltetrahydrofolate from plasma to the cytosol and regulation of intracellular folate concentration. It may limit the absorption of folic acid by the developing foetus, thus affecting the growth of the foetus [56]. In the current study, we did not observe an association between the studies SPNs of these genes and pregnancy or newborn complications.

The present study has several limitations such as a small sample size and short follow-up time. Moreover, all women included in this study were prescribed folic acid and vitamin B12 supplementation during pregnancy which possibly mask the association between one-carbon polymorphisms and pregnancy or newborn complications. Among the mother complications, a higher prevalence of GDM than the general population was found in this cohort. This is because pregnant women between 24 and 28 weeks of gestation who attended both the endocrinology and obstetrics departments were invited to participate in the study, and, in particular, the pregnant women who attended the endocrinology department mainly visited for metabolic problems, mostly GDM. Another limitation of this study could be that there are no metabolic data to assess the consequences of polymorphisms on the metabolism of single carbons, which limits the analysis of the significance of the associations. However, this may be the beginning of future studies with larger sample sizes and longer follow-up times that include both pregnant women as well as women with gestational desire to know the implication of these polymorphisms and their possible approach in the preconception stage.

## 5. Conclusions

In conclusion, our data show a high prevalence of genetic variants related to folic acid and vitamin B12 metabolic genes in pregnant women that may justify the difference in maternal and neonatal outcomes. This study warrants the need to perform further studies to elucidate whether knowing the prevalence of these polymorphisms on an individual basis and its association with the mother and newborn health may lead to personalised medicine with a nutritional assessment of vitamin intake.

## Figures and Tables

**Table 1:** Characteristics of the participants in this study.

Variables		Data	
***N***		149	
**Maternal age (years)**		34.7 ± 5.2	
**Pregestational BMI (kg/m^2^)**		26.4 ± 5.50	
**GDM (%)**		62.4	
**Socioeconomic status (% active)**		80.4	
**Type of gestation (%)**			
	**Spontaneous**	83.9	
	**Assisted**	15.1	
**Weight gain (kg)**		11.6 ± 5.84	
**Type of delivery (%)**			
	**Vaginal**	70.5	
	**Caesarean**	29.5	
	**Induced**	17.7	
	**Spontaneous**	82.3	
**Hospital stay (days)**		3.18 ± 1.37	
**GHT (%)**		2.0	
**Preeclampsia (%)**		3.4	
**Vitamin and mineral supplementation (%)**			
	**Vitamin complex**	81.2	
	**Iodine and folic acid**	13.4	
	**Folic acid**	5.4	
	**Iron**	55.0	
**Gestational week (wks)**		39.2 ± 1.9	
**Baby weight at birth (kg)**		3.18 ± 0.57	
**PTB (%)**		7.4	
**Neonatal ICU income (%)**		8.8	
**Baby sex (% women)**		48.3	
**SGA (%)**		3.4	
**AGA (%)**		89.9	
**LGA (%)**		6.7	
**Low birth weight (%)**		9.4	
**Macrosomia (%)**		6.7	

Data show mean ± standard deviation or frequency (%). *N*, number; BMI, body mass index; GDM, gestational diabetes mellitus; GHT, gestational hypertension; PTB, preterm birth; ICU, intensive care unit; SGA, small for gestational age; AGA, appropriate for gestational age; LGA, large for gestational age.

**Table 2:** Association between pregnancy outcomes and SNP genotypes of genes related to folate and cobalamin metabolism and transport in the study population.

Prevalence (%)	*SCL19A1* (rs1051266)			*CUBN* (rs11254363)			*CUBN* (rs1801222)			*MTR* (rs1805087)			*MTHFR* (rs1801133)			*MTHFR* (rs1801131)		
	TT	TC/CC	*p* Value	AA	AG/GG	*p* Value	AA	AG/GG	*p* Value	AA	AG/GG	*p* Value	CC	TC/TT	*p* Value	TT	TG/GG	*p* Value
**Samples**	28.15	71.85		59.26	40.74		52.59	47.41		74.81	25.19		39.26	60.74		47.41	52.59	
**Spontaneous gestation**	84.21	78.35	0.429	80.00	80.00	1.000	84.51	75.00	0.177	79.21	82.35	0.689	73.58	84.15	0.155	87.50	73.24	**0.035**
**Assisted fertilization**	7.89	18.56	0.075	15.00	16.36	0.836	11.27	20.31	0.158	16.83	11.76	0.460	20.75	12.20	0.208	9.38	21.13	**0.055**
**Vaginal delivery**	47.37	43.30	0.673	43.75	45.45	0.846	38.03	51.56	0.116	43.56	47.06	0.726	45.28	43.90	0.876	45.31	43.66	0.849
**Vaginal–instrumental delivery**	28.95	20.62	0.334	27.50	16.36	0.120	25.35	20.31	0.492	22.77	23.53	0.929	24.53	21.95	0.735	23.44	22.54	0.903
**Caesarean**	23.68	32.99	0.276	26.25	36.36	0.221	36.62	23.44	0.095	31.68	26.47	0.563	28.30	31.71	0.676	28.13	32.39	0.594
**Induced delivery**	18.42	15.46	0.693	16.25	16.36	0.986	19.72	12.50	0.261	15.84	17.65	0.815	18.87	14.63	0.535	20.31	12.68	0.243
**Spontaneous delivery**	81.58	83.51	0.798	83.75	81.82	0.777	80.28	85.94	0.390	82.18	85.29	0.672	79.25	85.37	0.380	79.69	85.92	0.350
**Preeclampsia**	2.63	4.12	0.698	3.75	3.64	0.977	2.82	4.69	0.625	2.97	5.88	0.567	0.00	6.10	**0.000**	4.69	2.82	0.625
**Preterm birth**	7.89	7.22	0.902	10.00	3.64	0.139	4.23	10.94	0.155	6.93	8.82	0.748	1.89	10.98	**0.013**	6.25	8.45	0.646
**Neonatal ICU income**	10.53	8.25	0.706	10.00	7.27	0.594	7.04	10.94	0.454	6.93	14.71	0.261	11.32	7.32	0.466	7.81	9.86	0.692

ICU, intensive care unit. AA, adenine-adenine genotype; AG, adenine-guanine genotype; GG, guanine-guanine genotype; TT, thymine-thymine genotype; TG, thymine-guanine genotype; CC, cytosine-cytosine genotype; TC, thymine-cytosine genotype; MTHFR, methylenetetrahydrofolate reductase; SLC10A1, reduced folate carrier (RFC1); MTR, methionine synthase; CUBN, cubilin. Numbers in bold show statistically significant differences.

## Data Availability

Data are available upon reasonable request from the corresponding author.
